# A systematic review of extended reality (XR) for understanding and augmenting vision loss

**DOI:** 10.1167/jov.23.5.5

**Published:** 2023-05-04

**Authors:** Justin Kasowski, Byron A. Johnson, Ryan Neydavood, Anvitha Akkaraju, Michael Beyeler

**Affiliations:** 1 Graduate Program in Dynamical Neuroscience, University of California, Santa Barbara, CA, USA; 2 Department of Psychological & Brain Sciences, University of California, Santa Barbara, CA, USA; 3 Department of Computer Science, University of California, Santa Barbara, CA, USA

**Keywords:** systematic literature review, assistive technology, virtual reality, augmented reality, blindness, low vision

## Abstract

Over the past decade, extended reality (XR) has emerged as an assistive technology not only to augment residual vision of people losing their sight but also to study the rudimentary vision restored to blind people by a visual neuroprosthesis. A defining quality of these XR technologies is their ability to update the stimulus based on the user’s eye, head, or body movements. To make the best use of these emerging technologies, it is valuable and timely to understand the state of this research and identify any shortcomings that are present. Here we present a systematic literature review of 227 publications from 106 different venues assessing the potential of XR technology to further visual accessibility. In contrast to other reviews, we sample studies from multiple scientific disciplines, focus on technology that augments a person’s residual vision, and require studies to feature a quantitative evaluation with appropriate end users. We summarize prominent findings from different XR research areas, show how the landscape has changed over the past decade, and identify scientific gaps in the literature. Specifically, we highlight the need for real-world validation, the broadening of end-user participation, and a more nuanced understanding of the usability of different XR-based accessibility aids.

## Introduction

In recent years, rapid technological advances have led to an increase in the number of assistive technology and electronic mobility aids for people with visual impairment (Butler, Holloway, Reinders, Goncu, & Marriott, 2021; Manjari, Verma, & Singal, 2020; Brulé, Tomlinson, Metatla, Jouffrais, & Serrano, 2020; Htike, Margrain, Lai, & Eslambolchilar, 2020). These assistive devices use various sensors (e.g., cameras, depth and ultrasonic sensors) to capture the environment and often apply computer vision and signal processing techniques to detect, recognize, or enhance text, people, and obstacles. While many devices convert visual information to tactile or audio information, the majority of people with visual impairment prefer to use their residual vision to observe the environment (Szpiro, Zhao, & Azenkot, 2016; Htike et al., 2020). People with no remaining light perception even have the option to receive a visual neuroprosthesis (Weiland, Liu, & Humayun, 2005; Fernandez, 2018), which is a device that electronically stimulates neurons in the visual pathway to restore a rudimentary form of vision.

One rapidly advancing technology being applied to low and prosthetic vision is extended reality (XR), which is an umbrella term that encompasses virtual reality (VR), augmented reality (AR), and other immersive mixed reality (MR) environments (Kardong-Edgren, Farra, Alinier, & Young, 2019). Generally speaking, VR refers to entirely simulated digital environments that block out outside sensory stimuli to increase the user’s sensation of verisimilitude (Coiffet & Burdea, 2003), whereas AR refers to manipulated or enhanced real-world environments, often through the use of visual overlays that provide supplementary or contextualizing information (Craig, 2013). In the context of visual accessibility, XR may be used either to benefit people with low vision via assistive technology (e.g., AR goggles that enhance the eyesight of people with low vision) or rehabilitation and training (e.g., VR applications and games for treatment of pediatric amblyopia) or to develop applications for sighted users that raise awareness about, and provide insight into, different visual impairments (e.g., VR applications that simulate the vision provided by a retinal implant). A defining property of these XR technologies is their ability to update the stimulus based on the user’s eye, head, or body movements. This would therefore include head-mounted devices such as prisms, goggles, and VR headsets but exclude other assistive technologies such as closed-circuit TV magnifiers and text-to-speech software.

To make the best use of these emerging technologies, it is valuable and timely to understand the state of this research and identify any shortcomings that are present. Previous reviews have highlighted a multitude of sensor-based technologies, ranging from smartphones (Manjari et al., 2020) to VR headsets (Htike et al., 2020; Aydmdoğan, Kavaklı, Şahin, Artal, & Ürey, 2021), which could be used to recognize commercial products (Machado, Veras, Aires, & Britto Neto, 2021), detect obstacles and reduce navigation time (Santos, Suzuki, Medola, & Vaezipour, 2021; Htike et al., 2020), or support social interactions (Qiu et al., 2022). These articles also pointed to several gaps in the literature and suggested potential avenues for future research. On the technology side, some studies suggested to use smart clothing (Santos et al., 2021) for nearby obstacle detection and to integrate devices with existing “Internet of Things” infrastructure (Machado et al., 2021). On the behavioral side, Kelly and Smith (2011) lamented that most studies in their review lacked methodological rigor. More recently, Brulé et al. (2020) highlighted the need for adequate quantitative empirical evaluation by involving appropriate end users in the design process. This sentiment was shared by Butler et al. (2021), who further highlighted the need to broaden application areas and ask for more in situ evaluation.

However, few systematic reviews have broadly summarized XR technology that uses *vision* as the primary feedback mechanism (Htike et al., 2020; Aydmdoğan et al., 2021). Whereas nonvisual feedback (e.g., via text-to-speech software or vibrotactile devices) is essential for people living with blindness, the majority of people with low vision prefer to use their residual vision to observe the environment (Szpiro et al., 2016; Htike et al., 2020). It is also valuable to take into account human factor considerations, such as the individual preferences and accessibility needs of people with different levels of residual vision, and cost, which remains an entry barrier even in developed countries (UNICEF, 2022).

The goal of this review is thus to summarize recent research in XR applications for people with blindness or low vision (BLV) and identify trends that can inform the development of future assistive technologies. This includes quantifying the number of studies, summarizing the major findings, identifying gaps in current practices, and making a number of specific recommendations for future research. Specifically, the goal was to answer a number of questions regarding the use of XR in BLV research:

• What are the main types of XR technologies used in BLV research?
• What experimental tasks are studied and how?
• What are key challenges or scientific gaps that researchers should focus on in the future?

## Methods

### Systematic review process

**Figure 1.**
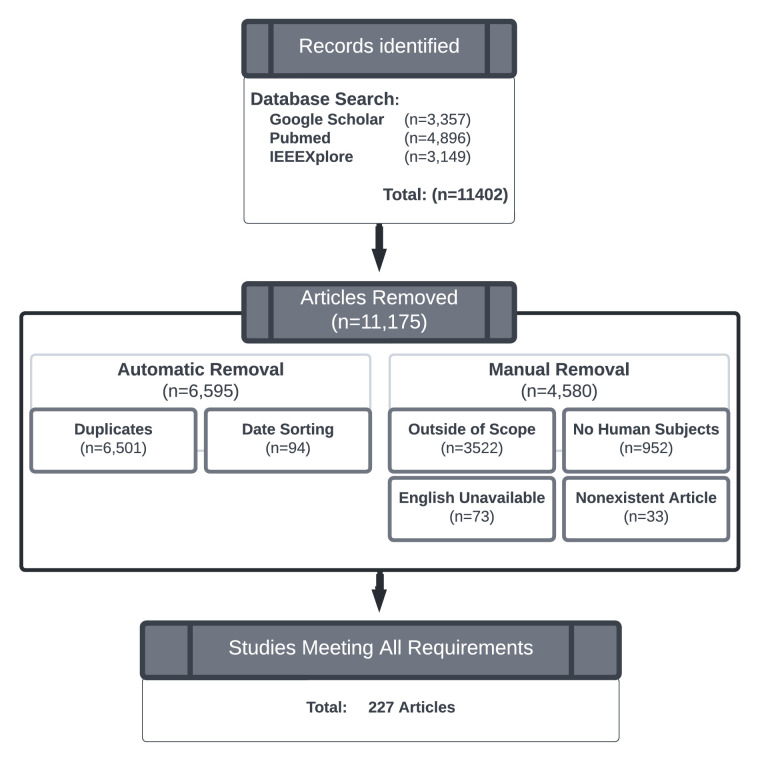
PRISMA flow diagram. The results from three databases (Google Scholar, IEEE Xplore, and PubMed) were searched to identify work that combined XR technology with low-vision research. After removing duplicates, improperly dated studies, and studies that did not involve human subjects research, we ended up with 227 articles to be included in the review.

In contrast to a traditional review, systematic reviews can provide a more complete and less biased picture of the type of work being undertaking in the field and point to key challenges moving forward (Mulrow, 1994). To help reduce bias and encourage a holistic review, we followed the PRISMA protocol (Page et al., 2021), which is a method for systematically searching databases with a list of keywords and documenting every step (Figure 1). This includes reporting the number of papers excluded from further analysis along with the reasons for exclusion.

To cover a large body of research independent of their publication venue, we searched three databases (Google Scholar, IEEE Xplore, and PubMed) on January 17, 2022. Each search included different keyword pairs (Table 1) designed to identify work that combines XR technology with low vision and accessibility research. Each database was searched with all allowable search parameters that did not result in a full-text search; that is, we searched the title alone with Google Scholar, title/abstract in PubMed, and title/abstract/author keywords in IEEE Xplore. This resulted in 11,402 matches across the three databases.

**Table 1.** Keyword combinations: Search terms used on Google Scholar, IEEE Xplore, and PubMed. Every “visual impairment” term was combined with all “extended reality” terms. “*” denotes the wildcard character.

Visual impairment		Extended reality		
“bionic vision”	“low*vision“	“AR”	“augment*”	“device*”
“prosthetic vision”	“retinal implant”	“display*”	“enhance*”	“head-mounted”
“retinal prosthesis”	“vis* aid*”	“immersive”	“mixed”	“reality”
“vis* loss”	“vis* impair*”	“simulat*”	“technolog*”	“wearable”

Due to the nature of searching multiple databases with numerous keyword combinations, a large number of duplicate articles were identified. All articles were imported into Zotero, which identified 6,501 duplicates and 94 other articles whose publication date preceded the year 2010.

The remaining 4,807 articles were reviewed by the research team and assessed for eligibility. A total of 4,580 papers were manually removed. The majority of these (*n* = 3, 522) were deemed outside the scope of the review as they presented a visual accessibility prototype that (even though it may operate on vision as an input modality) offered only nonvisual feedback to the user. While much has been written about the theoretical and technical aspects of accessibility technology, we specifically wanted to focus on studies that incorporated appropriate quantitative empirical evaluation, as suggested by Brulé et al. (2020) and Butler et al. (2021). Articles were therefore excluded if not an original work (e.g., review papers), if they solely proposed new technology without evaluating it on appropriate end users, or if they were focused on a survey about basic device use (i.e., “How often do you use your smart device to read text?”). Survey studies were included if they focused on participants’ perceived experience while using a specific technology. Smart devices (and their applications) were only included if they updated their visual augmentations in response to the user’s eye, head, or body movements. Furthermore, we removed 73 papers not available in English and 33 papers that could not be found online (most of these turned out to be manually entered citations on Google Scholar).

The remaining 227 studies, all of which were peer-reviewed, were included in the review.

### Interactive collection

The identified articles are available to the reader in three formats:

• as an interactive collection created with the free online platform “Litmaps” that can be accessed at https://app.litmaps.com/shared/map/CE0C5D29-8F18-4F2D-9866-0BE1EA4AF288, where visitors are able to inspect individual articles and see how they are connected to other articles in the collection;
• as a BibTeX file that can be used to cite references in LaTeX (see Supplementary Materials); and
• as an annotated spreadsheet that lists the type of devices used, level of immersion, the task performed, and the number of participants for each study (see Supplementary Materials), thus allowing the interested reader to deduce which studies were assigned to which of the subcategories introduced below.

**Figure 2.**
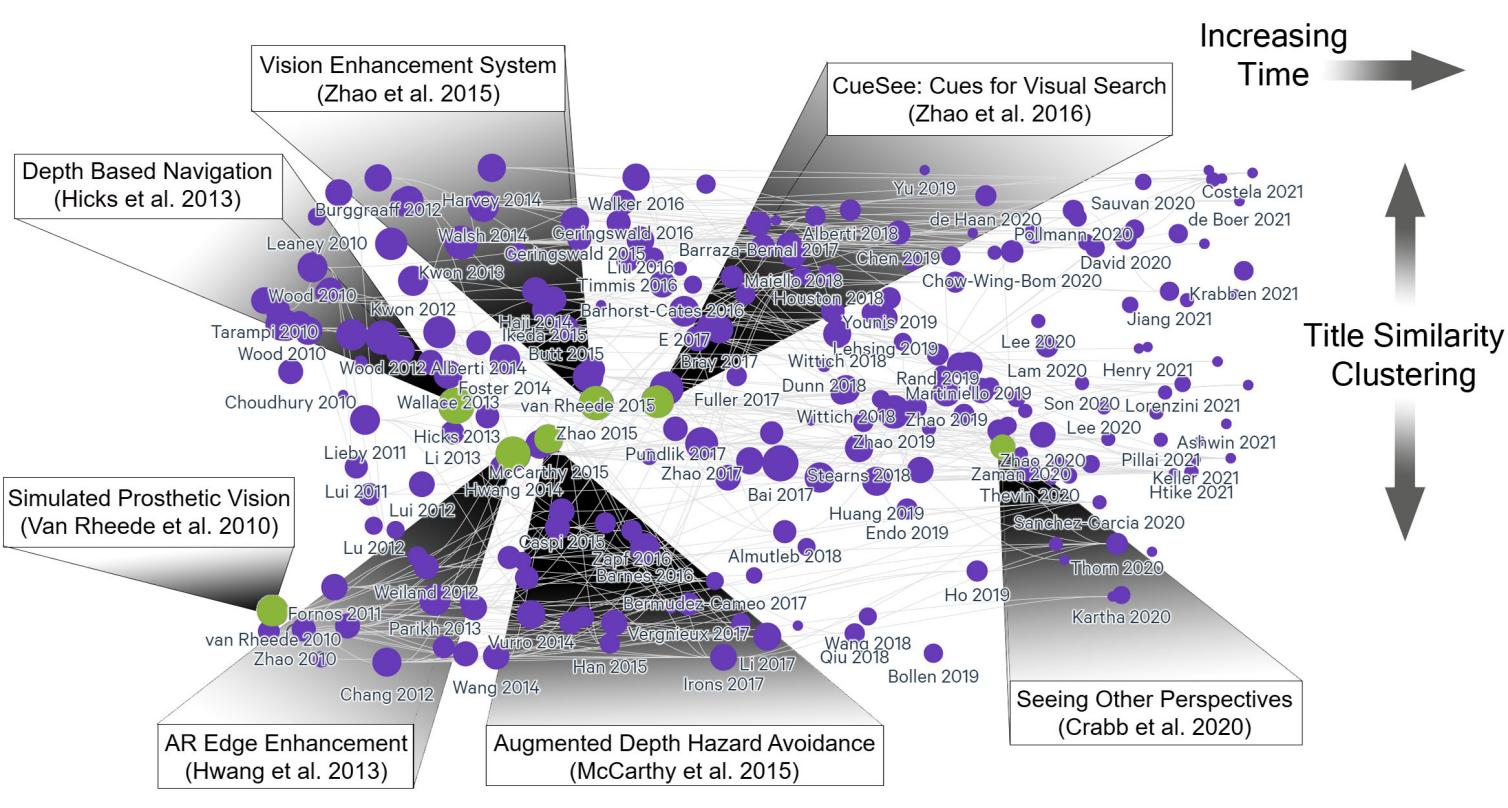
Corpus of identified articles presented chronologically from left to right. Each circle is a paper (size: number of citations), and some highly cited papers are highlighted with an inset illustration. Papers are organized vertically based on title similarity. An interactive version of the map is available at https://app.litmaps.com/shared/map/CE0C5D29-8F18-4F2D-9866-0BE1EA4AF288.

An example visualization of our interactive collection is shown in Figure 2, where each paper is represented by a circle whose size is proportional to the number of citations the paper received to date. The publication date increases moving left to right, and papers are spread over the y-axis according to how similar their titles are. To calculate title similarity, Litmaps uses Allen AI’s SPECTER model (Cohan, Feldman, Beltagy, Downey, & Weld, 2020), which projects the title of each paper into a 600-dimensional space before it is reduced to one dimension using UMAP. This view can be customized at the above URL, allowing visitors to cluster by keyword, title similarity, or citation count. A few select studies with a relatively large number of citations are highlighted in Figure 2.

## Research areas

**Figure 3.**
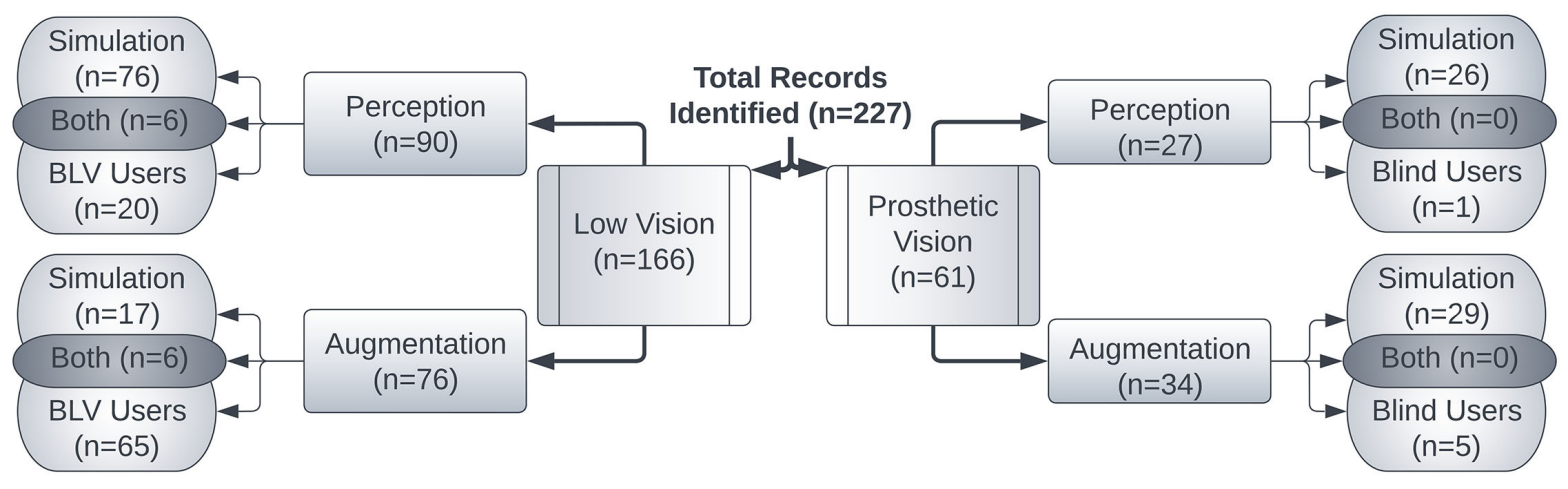
The 227 articles included in this review were manually assessed and categorized by (a) whether the end users were people with low vision (defined as having some residual light perception) or people who were totally blind (no light perception), (b) whether the article used XR technology to study visual perception and behavior or proposed a new XR augmentation technology, and (c) whether the article involved BLV end users, simulations of the relevant impairment condition, or both.

To get a better understanding of the research areas and applications covered by the corpus of identified papers, we inspected all 227 articles and hierarchically grouped them as follows:

• Level 1: Articles were categorized by whether end users were people with some residual light perception (*n* = 166; labeled “Low Vision” in Figure 3) *n* = 166) or blind people whose vision was restored with a neuroprosthesis (*n* = 61; labeled “Prosthetic Vision”).
• Level 2: Articles were classified either as “Perception” studies (if XR was used as a tool to study the visual perception and behavior of BLV end users) or as “Augmentation” studies (if the focus was on novel XR-based assistive devices or augmentation strategies).
• Level 3: Articles were categorized by whether participants were BLV end users (labeled “BLV Users” in Figure 3), sighted subjects viewing a low-vision simulation (labeled “Simulation”), or both.

As is evident from Figure 3, 73% of studies focused on low vision as opposed to blindness; however, within these two broad categories, there was a roughly equal focus on augmentation and perception. Interestingly, low-vision augmentation studies extensively involved low-vision participants (87% of studies), whereas all three other categories predominantly relied on computer simulations of the visual condition under study that would be presented to sighted participants. Roughly 7% of low-vision studies included both sighted participants (e.g., to evaluate a prototype using simulated low vision) and BLV (e.g., to validate their system on appropriate end users). This is in stark contrast to the prosthetic vision studies, none of which involved both sighted and blind participants.

To get a better understanding of the main types of XR technologies and experimental tasks used in BLV research, we screened every article in the collection to identify which XR display type was used, which experimental task was studied (and how), and whether BLV end users were involved (Table 2).

**Table 2.** Experimental tasks studied, extent of BLV end-user involvement, and XR display type used. Note that publications involve end users in multiple ways. If more than one task was studied or more than one display type used, the more rigorous metric was used. A, augmentation; P, perception.

		Low vision		Prosthetic vision		
		A	P	A	P	Total
**Experimental task**	Visual function testing	13	14	5	3	35
	Visual search/recognition	37	42	19	16	114
	Spatial cognition	26	34	10	8	78
**BLV end user involvement**	Prestudy qualitative assessment	30	16	0	0	46
	Evaluated BLV performance	52	18	5	1	76
	Poststudy qualitative assessment	20	8	2	1	31
**XR display type**	Monitors	10	31	3	8	52
	Handheld devices	14	0	0	0	14
	Nonelectronic wearables	8	39	0	0	47
	VR wearables	3	16	20	16	55
	AR wearables	41	4	11	3	59

All studies could be categorized as focusing on low-level visual function measurements (*n* = 47) such as acuity, contrast detection threshold, and orientation discrimination (e.g., Tatiyosyan, Rifai, & Wahl, 2020; Léné et al., 2020; Butt, Crossland, West, Orr, & Rubin, 2015; Almutleb, Bradley, Jedlicka, & Hassan, 2018); mid- to high-level visual function tasks (*n* = 111) such as visual search and object recognition (e.g., Walsh & Liu, 2014; Geringswald & Pollmann, 2015; Geringswald, Porracin, & Pollmann, 2016; Liu & Kwon, 2016); or high-level spatial cognition tasks (*n* = 72) such as wayfinding and obstacle avoidance tasks (e.g., Alberti, Horowitz, Bronstad, & Bowers, 2014; Zult, Allsop, Timmis, & Pardhan, 2019; Rand, Creem-Regehr, & Thompson, 2015; Murray et al., 2014) that require object recognition as well as locomotion.

We were also interested in knowing whether these studies were conducted with input or feedback from BLV end users by reporting at least one of the following:

• conduction of a prestudy qualitative assessment (e.g., surveys, questionnaires, or interviews) with BLV participants by the study authors, which was used to inform the design of a device/application;
• evaluation of perceptual or behavioral performance of the proposed simulation, device, or application with BLV end users; and
• conduction of a poststudy qualitative assessment (e.g., surveys or interviews) with BLV participants by the study authors, which was used to report about the usability of a device/application.

These numbers are summarized in Table 2. While 76 studies (33%) used BLV end users to evaluate performance, most prosthetic vision studies (95%) did not. Of the six studies that recruited bionic eye users, none consulted with BLV users about their information needs, and only two studies based their work on previous findings about the information needs of prosthesis users (Sadeghi et al., 2021; Rachitskaya et al., 2020). Additionally, while many studies used BLV participants, very few conducted poststudy qualitative assessments.

In terms of device types, VR wearables were the most popular device used (*n* = 75), followed by desktop monitors (e.g., combined with an eye tracker to provide gaze-contingent simulations of scotomas; *n* = 50), nonelectronic wearables (e.g., distortion goggles and lenses; *n* = 46), and AR wearables (e.g., AR smartglasses; *n* = 44). While all of these device types have been used in low-vision research, prosthetic vision studies have so far been restricted to monitors and VR/AR wearables. A detailed breakdown of studies by year and publication venue can be found in the Appendix.

Below we summarize the main research activities and findings following the hierarchical grouping introduced above (Figure 3). We highlight a few studies that we deemed representative of the corresponding subsection (often demonstrating a particularly impactful application of XR to low-vision research). We also aim to identify trends that can inform the development of future assistive technologies.

### XR for studying perception and behavior of people with low vision

Visual impairments such as age-related macular degeneration (AMD), glaucoma, and retinitis pigmentosa produce scotomas, that is, area(s) of the retina where the functioning of retinal cells is altered or diminished (Jones, Somoskeöy, Chow-Wing-Bom, & Crabb, 2020; Pollmann, Geringswald, Wei, & Porracin, 2020). Scotomas can lead to changes in visual function such as visual field loss, which may affect perceptual or behavioral performance (Jones et al., 2020; Pollmann et al., 2020).

Most of the studies in this category attempted to measure visual function either by recruiting people with low vision for testing a specific task (e.g., Miura et al., 2018; Hoppe, Anken, Schwarz, Stiefelhagen, & van de Camp, 2020) or by using low-vision simulations with sighted participants (e.g., Jones et al., 2020; Seitz, Maniglia, & Visscher, 2020).

#### XR for simulating the perception of people with low vision

Seventy-nine of the 90 identified low-vision perception studies (87.7%) relied on simulated low vision. An inexpensive means to simulate low vision for a sighted participant is the use of nonelectronic wearables, such as specially designed glasses, goggles, filters, and more (e.g., Kobashi, Kamiya, Shimizu, Kawamorita, & Uozato, 2012; Morris, Chaparro, Downs, & Wood, 2012; Scott, Atkins, Bentzen, & Barlow, 2012; Kanzler, Barth, Klucken, & Eskofier, 2016; Latham, Waller, & Schaitel, 2011). Modern alternatives include desktop displays or head-mounted displays that update the view based on where the user is looking (“gaze-contingent display”), which can be used to simulate specific eye conditions in real time (e.g., Kwon, Nandy, & Tjan, 2013; Seitz et al., 2020; Jones et al., 2020; also known as “altered reality,” Bao & Engel, 2019). While VR and AR headsets allow for similar experimental designs, researchers have direct control of the environment when using VR. The primary advantage of this approach is the ability to flexibly remove, add, or modify many different features of visual input.

**Figure 4.**
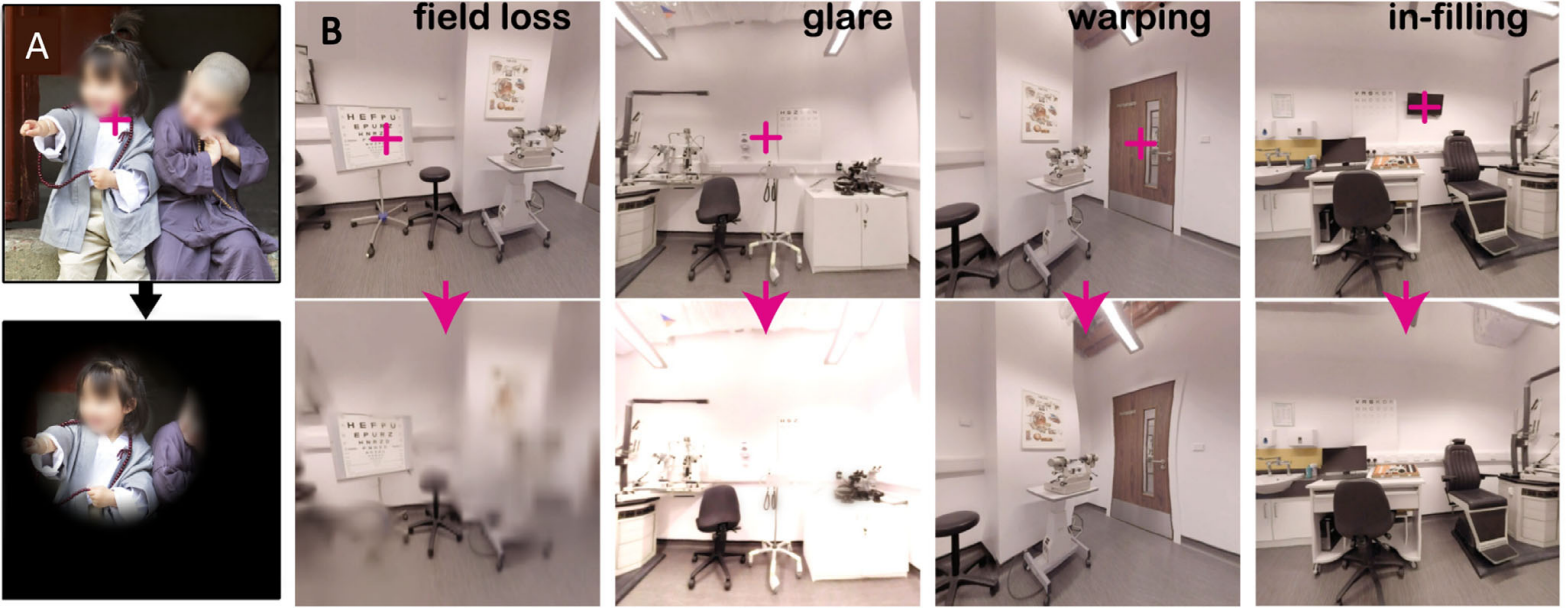
OpenVisSim conditions. (A) For a given fixation location (red cross), an example of simulated peripheral vision loss (“tunnel vision”) is shown. (B) Examples of visual changes associated with various low-vision conditions (reprinted under CC-BY from Jones et al., 2020).

Simulations are a valuable experimental tool for studying performance in tasks such as visual search (Addleman, Legge, & Jiang, 2021; Jones et al., 2020), face perception (Liu & Kwon, 2016; Tsank & Eckstein, 2017), reading (Huang et al., 2019; Latham et al., 2011), and navigation (Barhorst-Cates, Rand, & Creem-Regehr, 2019; Freedman, Achtemeier, Baek, & Legge, 2019; Zult et al., 2019). A prime example of this is OpenVisSim (Jones et al., 2020), which can track eye movements and simulate different gaze-contingent impairments in real time (Figure 4). To demonstrate the utility of OpenVisSim, Jones et al. (2020) simulated a central scotoma in VR (based on perimetric data from a person with glaucoma) and had sighted participants perform a visual search task with a Fove 0 headset and a mobility task with the HTC Vive. They demonstrated that the scotoma led to impaired performance in both tasks and found that a scotoma located in the upper visual field (inferior retina) led to worse performance, more eye movements, and more head movements.

However, the most commonly studied topic concerned the consequences of visual field loss on eye movements and associated behavior. It is well known that people with central visual field loss shift their oculomotor reference location from the fovea to an eccentric area known as the preferred retinal locus (PRL) (Bronstad, Bowers, Albu, Goldstein, & Peli, 2013). This happens gradually over time. Understanding PRL development and the behavioral consequences could potentially help people with low vision improve their oculomotor control in tasks such as reading and visual search (Kwon et al., 2013).

Many studies have thus trained sighted participants on a simulated scotoma with the help of the above-mentioned gaze-contingent displays, hoping that participants would develop a PRL. However, whereas some studies reported a shift in PRL with simulated low vision (SLV) in as fast as 3 hours (Kwon et al., 2013; Maniglia, Visscher, & Seitz, 2020; Seitz et al., 2020), others did not (David, Beitner, & Võ, 2020; Copolillo, Christopher, & Lyons, 2017; Almutleb et al., 2018). Longer explicit training times (i.e., 15 to 25 additional hours) have been shown to refine these effects to where oculomotor behavior was comparable to unimpaired controls (Kwon et al., 2013). Maniglia et al. (2020) conducted a systematic analysis of measures to understand how sighted participants can develop multiple PRLs and how individual participant re-referencing behavior is not consistent trial to trial even when trained. They found that roughly half of the participants exhibited saccadic re-referencing even without being instructed to do so (Maniglia et al., 2020). Kwon et al. (2013) showed that explicit training of a scotoma by highlighting estimated PRL locations effectively reduced the variance of fixation, much more than when participants were allowed to free-view the stimulus (i.e., the less variance, the more consistent their fixation locations).

Eye movements under SLV can vary drastically depending on how the impairment is presented to the participant (Kwon et al., 2013; David et al., 2020; Chow-Wing-Bom, Dekker, & Jones, 2020) and on what task is being studied (Tsank & Eckstein, 2017). For example, David et al. (2020) were able to show that saccade amplitude and fixation duration were significantly larger and longer with a simulated central scotoma, whereas the opposite effect was seen for a peripheral scotoma. Tsank and Eckstein (2017) showed that saccade patterns changed for an object-following and a visual-search task but not when identifying faces. In another study, McIlreavy, Fiser, and Bex (2012) were able to show that search times for targets and spatial distribution of gaze increased as the size of the simulated scotoma increased, while saccade amplitude and fixation duration remained unaffected.

While both Tsank and Eckstein (2017) and McIlreavy et al. (2012) provide insight into the effects of short-term impairment, it remains to be explored to which extent these simulations generalize to real people with low vision. Simulations of central vision loss with sighted participants have shown that PRLs can develop with training and that eye movements, while initially highly variable, can be refined over time (Liu & Kwon, 2016; Kwon et al., 2013; Maniglia et al., 2020; Seitz et al., 2020; Tsank & Eckstein, 2017). However, PRLs develop much quicker with sighted participants than with real AMD patients (Geringswald & Pollmann, 2015; Kwon et al., 2013), especially if the scotoma design includes a border and/or visual cueing for reference (Liu & Kwon, 2016; Seitz et al., 2020; Walsh & Liu, 2014). In contrast, people with AMD are often unaware of their scotoma location (Kwon et al., 2013).

#### XR for studying low-vision participants

To our surprise, only 20 of the 90 low-vision studies recruited participants with low vision (notable examples include Bowman & Liu, 2017; Powell, Powell, & Cook, 2020; Miura et al., 2018; Hoppe et al., 2020; Lin, Jan, Lay, Huang, & Chen, 2014; for the full list, please refer to the annotated spreadsheet in the Supplementary Materials), all of which were interested in studying how their oculomotor behavior differed from that of sighted people. Nine of 20 papers were interested in understanding how VR could be used to assess the behavior of BLV users. For example, Bowman and Liu (2017) trained low-vision participants in a street-crossing task. Four out of 12 participants were trained with real streets while the other 8 were trained with virtual streets using a three-screen VR projection system (subtending 168 × 35 degrees of visual angle). Both groups were tested on their street-crossing ability in real streets both before and after training. Before training, all participants demonstrated poor street-crossing skills (more than half of the responses were during “unsafe” times to cross). After training, over 90% of crossing responses were “safe.” Training with the VR system was comparable to training in real life, demonstrating how VR can be a powerful tool for practicing tasks that would otherwise be too dangerous or unfeasible within a laboratory setting (Bowman & Liu, 2017).

Lin et al. (2014) undertook the VR study with the largest sample size by recruiting 21 participants to perform a reading task while wearing a VR headset integrated with closed-circuit television magnification software. The head-mounted display with CCTV was used to obtain better depth of field and a higher modulation transfer function from the video camera. By sensing the parameters of the environment (e.g., ambient light level) and collecting the user’s specific characteristics, the system could make adjustments according to the user’s needs, which allowed participants to read more efficiently.

In sum, these studies highlight how VR headsets can be a tool for training and rehabilitation of improving reading skills for people with low vision.

#### Common limitations

An open question is to which extent low-vision simulations match the visual experience of real people with low vision. Many simulated low-vision studies involving sighted people base their simulations on crude approximations of a particular eye condition. For instance, to simulate a central scotoma, studies would often overlay a (rather salient) gray-filled circle over an image that would shift in sync with the participant’s saccades. In contrast, most people with AMD are unaware of their scotoma and also have different eye movements from sighted controls because of the scotoma (Kwon et al., 2013; Seitz et al., 2020). Recording of eye movements is therefore much more challenging for people with low vision since commercial devices are designed for nondisabled viewing. Furthermore, people with low vision are often much older than the sighted students typically recruited to participate in these simulation studies and have more experience using their residual vision for everyday tasks. It would therefore not be surprising if people with low vision showed differences in eye movement strategies and perceptual learning. Indeed, the results of previous SLV studies with respect to whether participants can learn to develop a preferred retinal locus remain mixed to date (e.g., David et al., 2020, vs. Kwon et al., 2013). In addition, sighted participants recruited for SLV typically ranged between 20 and 30 years of age, which is much younger than most people with central vision loss due to AMD (Klein et al., 2010). Perceptual and behavioral differences between sighted participants viewing low-vision simulations and real people with low vision may therefore be partially due to age difference (Yehezkel, Sterkin, Lev, & Polat, 2015).

A related limitation is the relative lack of BLV involvement in this line of research. While 75 of the 90 studies in this category referenced at least one previous study involving BLV (Table 2), we found only one study that grounded their simulation directly in clinical data (e.g., Jones et al., 2020). In addition, only a few studies aimed to assess the quality of their simulation by comparing performance to BLV participants. Future studies could thus work more directly with BLV and/or rehabilitation specialists, which may allow for a deeper understanding of how most simulations differ from the daily challenges that people with low vision have to deal with.

### XR for augmenting the residual vision of people with low vision

Another 33.5% of papers in our collection focused on the use of XR technology to augment and enhance the residual vision of people with low vision. This can range from handheld or wearables magnifying devices, to applications for smartphones and tablets, to wearable devices like head-mounted displays and smartglasses. Whereas VR allows for people with low vision to experience otherwise unsafe tasks in a controlled virtual environment, AR is better suited as a real-life visual accessibility aid (Gopalakrishnan, Chouhan Suwalal, Bhaskaran, & Raman, 2020), as it is allows for real-time interaction with an overlay of the real and digital world (similar to a hearing aid). Augmentation studies in this category focused on a variety of tasks, ranging from reading to face recognition (e.g., Costela, Reeves, & Woods, 2021b, 2021a; Calabrèse et al., 2018) and obstacle avoidance (e.g., Huang et al., 2019; Angelopoulos, Ameri, Mitra, & Humayun, 2019). Similar to the previous section, most of the studies in this category evaluated their augmentation prototype either directly on people with low vision (e.g., Calabrèse et al., 2018; Houston, Bowers, Peli, & Woods, 2018) or indirectly by using low-vision simulations with sighted participants (e.g., Hwang & Peli, 2014; van Rheede et al., 2015; Foster, Hotchkiss, Buckley, & Elliott, 2014). Some studies, like Zhao, Hu, Hashash, and Azenkot (2017), also used both.

#### XR for augmenting simulated low vision

A small number of studies in our corpus (*n* = 17) focused on digital image processing that may one day improve the behavioral performance of low-vision participants across different practical tasks. These visual augmentations were often added in real time to a gaze-contingent or a head-mounted display. For instance, low-level image manipulations such as increased text magnification and contrast were found to lead to faster reading speeds (Christen & Abegg, 2017), and enhancing the contours of faces and objects in a visual search task led to faster search times for older participants (Kwon et al., 2012). Other studies did not involve low-vision participants but instead added the visual augmentations on top of simulated low-vision conditions that were viewed by sighted participants. Christen and Abegg (2017) found that magnification was more beneficial for simulated blurry vision compared to a simulated scotoma, whereas contrast enhancement affected reading speed equally across simulated conditions. Similarly, temporal subsampling of an image (“image jitter”) was shown to improve peripheral acuity, word recognition, and facial emotion discrimination (Patrick, Roach, & McGraw, 2019; Watson et al., 2012).

Many of these studies aimed to understand how smartglasses could be used to benefit people with low vision (e.g., Hwang & Peli, 2014; Huang et al., 2019; Zhao, Hu, et al., 2017). See-through head-mounted displays such as Google Glass and Microsoft Hololens are systems that are commercially available for testing. Hwang and Peli (2014) measured contrast sensitivity for two conditions with three sighted participants: with or without AR edge enhancement and with or without a heavy diffuse film (Hwang & Peli, 2014). The enhancement being tested was the Laplacian edge detection method, where a positive method enhanced edges while a negative method enhanced the surrounding of edges. Contrast sensitivity thresholds had improved with the enhancement method (Hwang & Peli, 2014). Huang et al. (2019) tested 24 sighted participants on a navigation task with a voice-based sign reading application for the Hololens. All participants wore goggles modified with occlusion foils during the task to simulate reduced acuity. Results indicated that participants walked more slowly and took more time with the sign-reading application. Overall, participants walked on more direct paths and were more confident with the application.

#### XR for studying augmentations for low-vision participants

Although results from the above simulation studies are notable, the ultimate goal of an XR accessibility aid should be to improve the residual vision of real people with low vision. In line with this goal, the majority of studies in this category thus evaluated their prototypes on appropriate end users.

**Figure 5.**
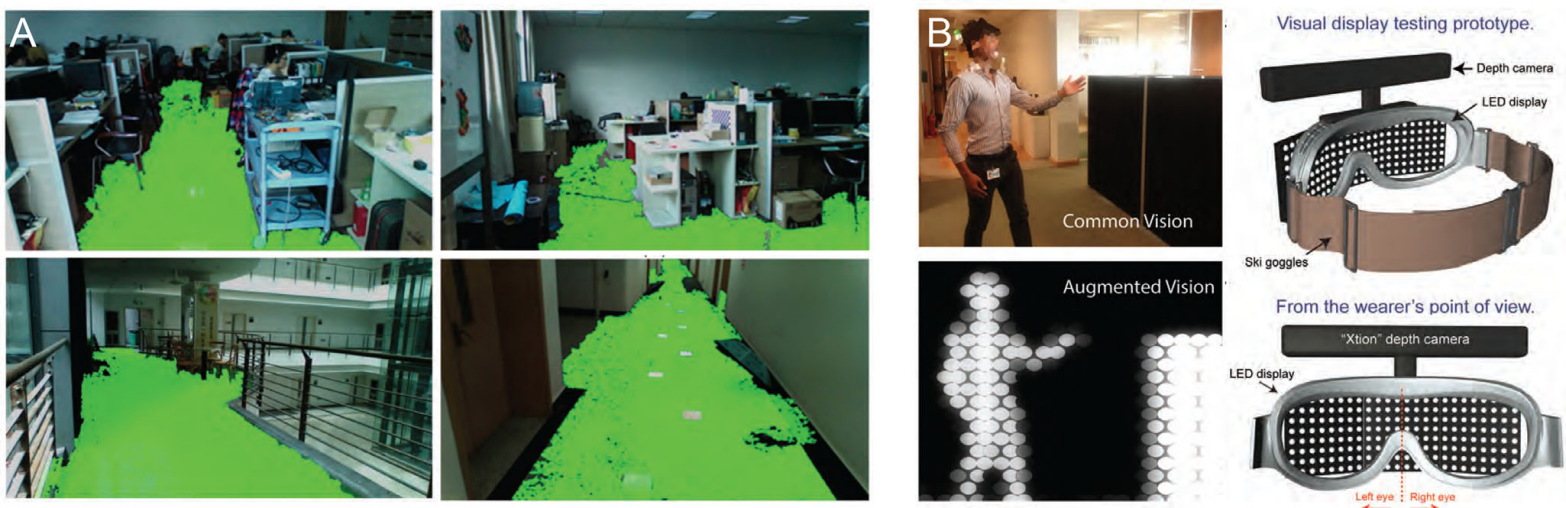
Examples of augmented reality in a head-mounted display. (A) “RealSense” is able to detect and highlight the traversable area in a variety of structured indoor environments (reprinted under CC-BY from Yang et al., 2016). (B) A depth camera designed for detecting people and obstacles while walking (reprinted under CC-BY from Hicks et al., 2013).

Several groups have explored how XR may benefit people with low vision perform different activities of daily living, such as navigating in unfamiliar environments or identifying objects of interest. For instance, “RealSense” (Yang, Wang, Hu, & Bai, 2016) is an AR application that automatically detects and highlights the traversable area in both indoor and outdoor environments (Figure 5A). Rather than highlighting nearby obstacles, the authors argued that highlighting the traversable area would better allow participants to plan their paths around obstacles. A related idea was presented by Zhao, Kupferstein, Rojnirun, Findlater, and Azenkot (2020), who used AR smartglasses to annotate the natural scene with “turn-by-turn” instructions for wayfinding akin to a car navigation system. Using a control condition that provided only audio feedback, the authors were able to demonstrate that low-vision participants made fewer mistakes and walked faster when using visual feedback.

Houston et al. (2018) tested the ability of people with visual field loss to navigate a virtual mall while wearing specially designed glasses (peripheral prisms) that could expand the binocular visual field by up to 40 degrees. Twenty-four participants were asked to report obstacles and pedestrians while navigating the virtual mall. Interestingly, the detection of hazards on the same side of the visual field defect improved significantly for most participants, even without training (Houston et al., 2018). In another study, participants with various diagnosed forms of visual impairment were able to safely complete a stair navigation task with the help of an AR headset designed to highlight stair edges (Zhao, Kupferstein, Castro, Feiner, & Azenkot, 2019).

Few studies in our bibliography focused on improving reading. A notable exception is “ForeSee” (Zhao, Szpiro, & Azenkot, 2015; Zhao, Kupferstein, et al., 2019), an AR application that uses a combination of general low-level image enhancement methods (e.g., magnification, contrast enhancement, edge enhancement) for reading text in near- and far-distance viewing conditions. The benefit of “ForeSee” is that users can choose any of the enhancements presented in two display modes (full view or windowed), customizing the viewing experience as they see fit. Magnification and the windowed mode were the methods most preferred by participants, but the ability to use a combination of enhancements in real time was reported to have the strongest influence on a user’s viewing experience (Zhao et al., 2015).

Assistive devices are designed to help users see details (such as in reading), but often these devices are not designed to assist in other visual tasks. Enhancement of visual search was the main motivation for developing “CueSee”: an AR application to enhance recognition of targets with the help of five different attention enhancement cues, including magnification, color enhancement, flashing bounding boxes, and rotation. The researchers designed a search task with a mock grocery shelf in which different items were marked using AR tags (“Chilitags”; https://github.com/chili-epfl/chilitags), though a future iteration of the application may rely on real-time object recognition. Participants identified items on a grocery shelf significantly faster and more accurately using CueSee than without it. More importantly, participants preferred the CueSee enhancements over traditional cues (Zhao, Szpiro, Knighten, & Azenkot, 2016).

Whereas most studies focused on enhancing a user’s residual vision, others built custom head-mounted displays to simplify the visual scene. A notable example is the work by van Rheede et al. (2015), which built a headset integrated with an infrared depth camera to create a depth map, which was relayed to the user as a grayscale map: The closer the obstacle, the brighter the representation on the display (Figure 5B). Participants were then instructed to avoid foam obstacles while navigating a hallway; 6 of the 11 participants completed the obstacle course without any collisions.

Lastly, with XR systems becoming more common in low-vision research, Zhao, Cutrell, et al. (2019) asked how the user experience in VR itself may be improved. To address this question, they developed “SeeingVR” (Zhao, Cutrell, et al., 2019), a set of 14 visual enhancement tools that include digital magnification, brightness and contrast controls, edge enhancement, peripheral remapping, text augmentation, depth measuring, and text to speech, which can be overlaid post hoc onto any existing VR application. When asked to use a virtual keyboard, navigate an options menu on the screen, search for an object, or shoot a moving target while wearing a HTC VIVE, 11 participants with low vision completed the tasks much faster and more accurately with SeeingVR than without the overlay application (Zhao, Cutrell, et al., 2019). Moreover, users reported finding VR more enjoyable when using SeeingVR, making this work a promising first step toward the design of general accessibility standards for VR.

Although most studies in this subcategory focused on technology development, some also assessed the usability of the proposed XR systems. One study reported that most people with low vision preferred a compact device similar to a regular pair of glasses with buttons for inconspicuous interactions (Hoogsteen, Osinga, Steenbekkers, & Szpiro, 2020). Another study pointed to the portability of a head-mounted system paired with a smartphone as a camera as the preferred form factor for a reading aid (Stearns, Findlater, & Froehlich, 2018). The ForeSee work (Zhao et al., 2015) highlighted the need to give users the option to choose from several enhancement modes. Another AR study found that alphanumeric representation of information may be better for those with relatively higher acuity, whereas symbolic representation may be better suited for those with worse acuity (Lang, Schmidt, & Machulla, 2020). Audio feedback was generally liked by participants as well (Zhao et al., 2020): Although some participants preferred audio feedback because of shorter learning curves, all participants reportedly wanted to combine visual and audio features to refine their wayfinding experience.

#### Common limitations

Although most works evaluated their XR prototype on low-vision participants as a proof of concept, relatively few studies recorded participant feedback after the study was conducted (e.g., Zhao, Cutrell, et al., 2019, 2020; Min Htike, Margrain, Lai, & Eslambolchilar, 2021; Htike, 2020). However, this may be an important step toward designing more usable accessibility aids that are sensitive to the information needs of people with blindness or low vision (Htike et al., 2020). For instance, Williams, Galbraith, Kane, and Hurst (2014) compared sighted and blind navigation and found that both groups understand navigation differently, leading sighted people to struggle in guiding blind companions. In addition, people with blindness or low vision use a combination of devices and technology to complement their existing orientation and mobility skills (Williams et al., 2014), which may lead to a wide variety of navigation styles (Ahmetovic, Guerreiro, Ohn-Bar, Kitani, & Asakawa, 2019; Htike et al., 2020). In the future, further collaboration between researchers and end users could benefit from device design by augmenting the visual environment based on user-specific needs.

It is interesting to note that, despite demonstrating an improvement in task performance, many studies reported an increase in trial completion time (e.g., van Rheede et al., 2015; Zhao, Hu, et al., 2017), often linked to slower walking speeds or longer search times. While this may indicate that participants were more careful, it could also indicate increased hesitation or lower confidence when using VR and AR controls. In addition, individual differences in visual function (i.e., acuity, thresholds, etc.) could have more effects on performance (Lang et al., 2020).

### XR for studying perception and behavior of people with prosthetic vision

XR technology has been used not only to augment the vision of people with low vision but also to study the rudimentary vision restored to blind people by a visual neuroprosthesis (“bionic eye”; 15.0% of papers in our collection). Similar to conventional AR headsets, visual prostheses typically contain an external camera mounted on a pair of glasses that is used to relay the visual scene to the user (Fernandez, 2018). However, in contrast to conventional AR headsets, visual prostheses also consist of an implantable microstimulator (implanted in the eye or the visual cortex), which decodes the visual information and electrically stimulates neurons in the visual pathway to evoke visual percepts (“phosphenes”). Existing bionic eyes generally provide an improved ability to localize high-contrast objects and perform basic orientation and mobility tasks (e.g., Stronks & Dagnelie, 2014). While this could be considered a rudimentary form of AR on its own, a good number of studies used VR to simulate the perception produced by these devices.

#### XR for simulating prosthetic vision

To investigate functional recovery and experiment with different implant designs, researchers have been developing XR prototypes that rely on simulated prosthetic vision (SPV). The classical method relies on sighted subjects wearing a VR headset, who are then deprived of natural viewing and only perceive phosphenes displayed in the head-mounted display. This viewing mode has been termed “transformative reality” (Lui, Browne, Kleeman, Drummond, & Li, 2011, 2012) (as opposed to “altered reality,” which is typically used to describe simulated low-vision approaches; Bao & Engel, 2019). This allows sighted participants to “see” through the eyes of the bionic eye user, taking into account their head and/or eye movements as they explore a virtual environment (Kasowski, Wu, & Beyeler, 2021).

One application of SPV is assessing low-level visual function, and three studies were placed in this category. These studies focused on aspects like phosphene size (Lu et al., 2012) and shape (Cao, Li, Lu, Chai, & Wang, 2017) by varying stimulus and model parameters. Stimuli for these tasks are typically presented on a monitor (Lu et al., 2012), via AR glasses (Caspi & Zivotofsky, 2015), or in a head-mounted display (Cao et al., 2017). One prominent example (Caspi & Zivotofsky, 2015) used sighted volunteers to complete a Landolt-C visual acuity task using SPV. Participants wore custom AR glasses to view webcam input that was converted to an 8 × 8 pixel image, meant to represent the limited resolution of current retinal implants. The authors found that well-performing participants developed similar strategies to those employed by real prosthesis users, such as scanning the image using strategic head movements. Interestingly, by utilizing head movements, the participants were able to surpass the theoretical acuity limit. This phenomenon had previously been identified in real prosthesis users (Humayun et al., 2012), and the authors hypothesized it was the accumulation of information over time. By utilizing a simple low-level visual function XR experiment, Caspi and Zivotofsky (2015) were able to confirm this hypothesis.

**Figure 6.**
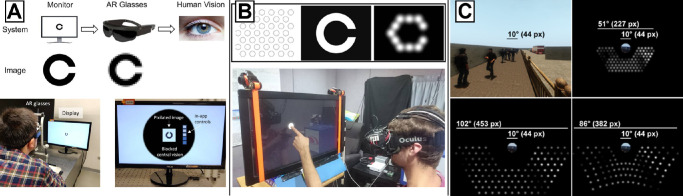
Examples of augmented reality systems used to simulate prosthetic vision with sighted participants. (A) AR glasses for mimicking the prosthetic vision seen by a participant with geographic atrophy (reprinted under CC-BY from Ho et al., 2019). The front camera of the AR glasses captured the video stream, while custom software preloaded on the glasses adjusted the video quality to mimic prosthetic vision (*bottom*). (B) AR system to evaluate the benefit of gaze compensation on hand–eye coordination (reprinted under CC-BY from Titchener, Shivdasani, Fallon, & Petoe, 2018). Phosphenes were rendered as Gaussian blobs (*top*). Participants wore a simulated prosthetic vision headset that included a front-facing camera, head motion tracker, and eye tracker (*bottom*). (C) Simulated prosthetic vision in retinitis pigmentosa. Residual vision covers the central 10^*o*^ field of view, and simulated electrode arrays provide bionic vision in the degenerated periphery (reprinted under CC-BY from Zapf, Boon, Matteucci, Lovell, & Suaning, 2015).

The majority of studies in this section focused on slightly more complex tasks such as letter (Zhao et al., 2011), word (Fornos, Sommerhalder, & Pelizzone, 2011), face (Denis, Jouffrais, Vergnieux, & Macé, 2013; Chang, Kim, Shin, & Park, 2012), and object recognition (Zhao et al., 2010; Wang, Sharifian, Napp, Nath, & Pollmann, 2018; Macé, Guivarch, Denis, & Jouffrais, 2015). This group of studies had the highest average number of subjects (μ = 21.06 ± 12.34) when compared to other areas of SPV studies. In most setups, participants would view SPV stimuli in a conventional VR headset, but a large portion used a monitor with some sort of eye tracking. Surprisingly, although the majority of the tasks used head-mounted displays, none of the studies allowed for a fully immersive experience that would allow the subject to walk around and interact with the environment. Studies in this category primarily used SPV to study basic behavior in these tasks, but some studies also used these tasks to focus on another behavior. One example is the work by Sanchez Garcia, Martinez-Cantin, Bermudez-Cameo, and Guerrero-Campo (2020). In this work, participants were tasked to find and recognize objects in a scene with different fields of view (20°, 40°, or 60°) and number of phosphenes (200 or 500). The authors showed counterintuitive results, with a higher field of view resulting in significantly worse performance and longer recognition times. However, they argued that phosphene density may be more important for object recognition than field of view, which is consistent with earlier findings (van Rheede, Kennard, & Hicks, 2010). Ho, Boffa, and Palanker (2019) relied on AR smartglasses to simulate the artificial vision provided by the PRIMA subretinal implant (Lorach et al., 2015) (Figure 6A). This device was developed for people with geographic atrophy as commonly experienced with AMD, where vision is first lost in the macula. To simulate this, the authors needed to combine SPV in the macula and natural vision in the periphery. The authors accomplished this by using AR smartglasses with black tape occluding the central field of view so only the LED overlay was visible in this area. With this setup, they were able to make testable predictions about the visual acuity to be expected from PRIMA (Lorach et al., 2015), which is currently in clinical trials.

Lastly, a number of studies focused on spatial cognition tasks, such as obstacle avoidance (Zapf, Boon, Lovell, & Suaning, 2015, 2016; Endo et al., 2019) and wayfinding (Vergnieux, Macé, & Jouffrais, 2014). By design, these tasks require a more immersive setup that allows for the incorporation of head and eye movements as well as locomotion (Kasowski & Beyeler, 2022). Most of these studies incorporated a fully immersive design for their task, although a few used VR headsets but required their subjects to sit/stand in place and instead use a keyboard/controller to move. The majority of tasks were simply “proof-of-concept” experiments showing that users were able to navigate effectively with SPV. A notable example is Zapf, Boon, Matteucci, et al. (2015), who simulated tunnel vision as typically encountered during retinitis pigmentosa by restricting participants to their central 10° field of view in a virtual environment. Eleven participants completed a variety of tasks consisting of low-lying obstacle circumvention (avoiding traffic cones), static/moving pedestrian avoidance (navigating a corridor with stationary/moving virtual characters), and path following (following a path through parked cars). The authors then wanted to know how behavioral performance might improve when visual cues in the (presumed degenerated) periphery were provided by a simulated retinal implant (see Figure 6). Although behavioral performance improved for avoiding low-lying obstacles and following paths, the simulated prosthetic vision in the periphery could not help participants avoid stationary head-level targets.

#### XR for studying prosthesis users

Rachitskaya et al. (2020) was the only study to use XR for visual rehabilitation of real bionic eye users. It was also the only study to mention consultation with the BLV community during development, having utilized an interdisciplinary team that incorporated ophthalmologists and rehabilitation specialists. Since there currently is no standardized procedure for vision rehabilitation across different Argus II implantation centers, Rachitskaya et al. (2020) developed a Computer-Assisted Rehabilitation Environment (CAREN), which consists of a motion capture system, control software with a 180° curved projection screen, a motion platform, and a treadmill. Participants donned a harness, had access to handrails on the treadmill, and were accompanied by a physical therapist. After using CAREN twice a week for 4 weeks, participants showed significant improvements in walking speed and object localization, demonstrating that immersive technology may provide a solution for the standardization of effective rehabilitation approaches to augment bionic eye performance.

#### Common limitations

An open question is to which extent prosthetic vision simulations match the visual experience of real prosthesis users. Similar to SLV research, many SPV studies base their simulations on crude approximations of prosthetic vision, assuming that each electrode acts as a small independent light source that produces a distinct focal spot of light (Dobelle, 2000). However, a growing body of evidence suggests that the vision generated by current visual prostheses is “fundamentally different” from natural vision (Erickson-Davis & Korzybska, 2021), with interactions between implant technology and the neural tissue degrading the quality of the generated prosthetic vision (Fine & Boynton, 2015; Beyeler et al., 2019). Only 4 out of 27 studies in this category incorporated a great amount of neurophysiological detail into their setup (Josh, Mann, Kleeman, & Lui, 2013; Vurro, Crowell, & Pezaris, 2014; Wang et al., 2018; Thorn, Migliorini, & Ghezzi, 2020), only 2 of which (Wang et al., 2018; Thorn et al., 2020) relied on an established and psychophysically evaluated model of SPV. In addition, the level of immersion offered by most SPV studies was relatively low, with many studies simply presenting simulated stimuli on a screen without taking into account the participant’s gaze. However, most current prostheses provide a very limited field of view; for example, the artificial vision generated by Argus II (Luo & da Cruz, 2016), the most widely adopted retinal implant thus far, is restricted to 10 × 20 degrees of visual angle. This requires users to scan the environment with strategic head movements while trying to piece together the information (Erickson-Davis & Korzybska, 2021). It is therefore unclear how the findings of most SPV studies would translate to real bionic eye users.

Additionally, only a single study in our collection worked with real bionic eye users or rehabilitation specialists (Rachitskaya et al., 2020). XR may offer a unique method for safe training and rehabilitation but is severely underutilized in comparison to research on XR for low vision.

### XR for augmenting prosthetic vision

Another 15% of papers in our collection focused on the use of XR technology to augment and enhance prosthetic vision, either through simulations (*n* = 29) or the use of peripherals and extra sensors to extract visual scene information for real bionic eye users (*n* = 5).

#### XR for augmenting the visual scene using simulated prosthetic vision

A popular trend for SPV is utilizing novel augmentation strategies to aid scene understanding. One approach is using computer vision to enhance certain image features or regions of interest, at the expense of discarding less important or distracting information. Various studies have explored strategies based on visual saliency (e.g., Parikh, Itti, Humayun, & Weiland, 2013), background subtraction and scene retargeting (e.g., Li, Zeng, et al., 2018), and depth mapping to highlight nearby obstacles (e.g., Lieby et al., 2011; McCarthy, Walker, Lieby, Scott, & Barnes, 2015; Kartha et al., 2020). For instance, McCarthy et al. (2015) used an RGB-D camera mounted on a pair of AR glasses to augment the visual scene with depth information. The study used a custom-augmented reality setup utilizing a head-mounted display with an attached stereo camera. The images from the camera were sent to a laptop on the participant’s back and were processed into a simplified model of bionic vision using a pixel display with 20 phosphenes. Among their tested image-processing strategies, augmented depth proved the most effective at highlighting hazards in the path; this mode modified the depth information from the stereo cameras to detect objects while simultaneously removing the ground from the scene. They found a significantly reduced rate of collisions, even in the presence of low-contrast trip hazards. The same research group evaluated their findings with real bionic eye users and found similar performance increases (Barnes et al., 2015). This specific example shows how SPV can be used to rapidly examine possible augmentations and lead to enhancements for real prosthesis users.

The majority of SPV studies in this category used monitors, VR headsets, and AR glasses to improve performance on recognition tasks, such as identifying faces (Chang et al., 2012; Wang et al., 2014; Irons et al., 2017), text (Denis, Jouffrais, Mailhes, & Mace, 2014; Paraskevoudi & Pezaris, 2021), and objects (Li, Zeng, et al., 2018; Wang et al., 2016). For instance, a number of studies (Chang et al., 2012; Wang et al., 2014; Irons et al., 2017) highlighted through simulations that face caricaturing, where prominent facial features are highlighted or enhanced, can improve face recognition for sighted subjects viewing SPV. Studies focused on recognition applied various enhancements, including contrast enhancement (Chang, Kim, & Park, 2010), saliency algorithms (Li, Su, et al., 2018; Wang et al., 2016), edge/foreground extraction (Han, Li, Lyu, Zeng, & Chai, 2015; Lui et al., 2011), and facial landmark extraction (Bollen, Güçlü, van Wezel, van Gerven, & Güçlütürk, 2019).

Eight SPV studies focused on spatial cognition tasks, including wayfinding (Vergnieux, Macé, & Jouffrais, 2017; van Rheede et al., 2010), obstacle avoidance (McCarthy et al., 2015), and environmental search (Parikh et al., 2013). For instance, van Rheede et al. (2010) used gaze-contingent SPV to measure acuity, object recognition, and mobility. They found that using a region-of-interest view improved acuity while a wide field of view was better for mobility, highlighting the use of testing multiple forms of enhancement with various tasks. In a similar manner to the spatial behavioral SPV studies in the previous section, the majority of augmentation SPV studies also used VR headsets, with some studies using AR smartglasses (Weiland, Parikh, Pradeep, & Medioni, 2012; McCarthy et al., 2015; Parikh et al., 2013). Most of the studies were fully immersive, but two used VR headsets without positional tracking (Vergnieux et al., 2017, 2014). Out of these eight studies, only one study used eye tracking (van Rheede et al., 2010) or presented monocular stimuli (Parikh et al., 2013). These studies also suffered from relatively low subject counts ranging from 4 to 19 subjects (μ = 10.75 ± 4.62).

The remaining three simulation studies in this category focused on low-level visual function (Bermudez-Cameo, Badias-Herbera, Guerrero-Viu, Lopez-Nicolas, & Guerrero, 2017; Al-Atabany, Al Yaman, & Degenaar, 2018; Titchener et al., 2018). Two of these studies used AR for enhancing an SPV scene: Al-Atabany et al. (2018) using infrared (IR) overlays for counting people/actions in a scene and Bermudez-Cameo et al. (2017) using RGB-D cameras for depth overlays in a target localization task. The third study, Titchener et al. (2018), used a VR headset with eye tracking to study the effects of gaze in a simulated retinal prosthesis (Figure 6B). Seven sighted subjects performed a target localization-pointing task under uncompensated and gaze-compensated SPV. Not surprisingly, subjects had a significantly smaller pointing error using gaze compensation (Titchener et al., 2018). This simulation result was also confirmed with real bionic eye users (Caspi et al., 2018).

#### XR for studying augmentations for prosthetic vision

While a bionic eye is technically itself a technology that augments vision, several studies focused on augmentation strategies outside the basic stimulation patterns of the device. This includes thermal imaging (Zagar & Baggarly, 2010; He, Huang, Caspi, Roy, & Montezuma, 2019), audiovisual cross-modal mapping (Stiles, Patel, & Weiland, 2021), and depth detection with object segmentation (Kartha et al., 2020). For example, Sadeghi et al. (2021) tested the ability of bionic eye users to perform a series of practical tasks (e.g., identifying hot objects, estimating the distance to a nearby person) while using a thermal camera. The study highlighted improved performance across all tested tasks, including tasks where thermal integration would be considered an obvious benefit (e.g., identifying the closer side of a hot cup, identifying a missing bowl that was heated), but also tasks such as identifying when people were on an escalator and, additionally, determining whether an escalator was moving toward or away from them. In another study by Kartha et al. (2020), Argus II users completed various tasks with a distance-filtered input. In this study, the removal of distant clutter was able to improve participant performance across a variety of tasks, including size, depth, and walking direction discrimination. Although behavioral performance was often still close to chance levels, these results are promising and present the possibility for more advanced augmentation methods to be useful in the future.

#### Common limitations

Similar to the previous section, SPV studies that augmented prosthetic vision relied on crude approximations of the visual experience that cannot explain the perceptual distortions encountered by real bionic eye users (Beyeler et al., 2019; Erickson-Davis & Korzybska, 2021). Although there is no shortage of publications that demonstrate a proof-of-concept augmentation strategy, more research is needed to compare these approaches side-by-side (Han, Srivastava, Xu, Klein, & Beyeler, 2021), and only a few studies discussed the usability aspects of their proposed technology (Sadeghi et al., 2021; Kartha et al., 2020). Additionally, only 6 out of 29 SPV studies allowed participants to move around in an immersive way. Typical real-life scenarios cannot be mastered while stationary, and future studies may benefit from allowing participants to move around their environment. Many studies used SPV to assess the benefit of their proposed technology, but very few used models based on neuroscience, considered gaze, or presented monocular stimuli.

Because the involvement of real bionic eye users remains limited (500 implantees worldwide) and challenging (e.g., constant assistance, increased setup time, travel cost), it is not surprising that most behavioral studies that recruited real prosthesis users were reported with a relatively small sample size (one to five participants). While XR technology in combination with SPV may provide a more cost-effective alternative to prototyping novel augmentation strategies (Kasowski et al., 2021), future studies should consider a more direct comparison between their theoretical predictions and the visual experience reported by real bionic eye users (Beyeler et al., 2019; Erickson-Davis & Korzybska, 2021).

## Discussion

### The main types of XR technologies used in BLV research

As we set out to discover the prevalence of different XR technologies in BLV research, we found that VR wearables were by far the most popular device type among the studies in our corpus, prevalent in both low-vision and prosthetic vision research (Table 2). The most commonly used VR headsets included the HTC Vive, Fove 0, and Oculus Rift (e.g., Hoppe et al., 2020; Zhao, Cutrell, et al., 2019; Jones et al., 2020; Chow-Wing-Bom et al., 2020; Kvansakul, Hamilton, Ayton, McCarthy, & Petoe, 2020). Interest in these devices has been more or less constant over the past decade but has seen a recent increase in 2020–2021 (see Supplementary Materials). VR devices have the advantage of allowing researchers to fully control the visual stimuli presented to the participants, which can make for a flexible testbed for prototypes of near-future visual accessibility aids (Hoppe et al., 2020; Zhao, Cutrell, et al., 2019). They also offer a safe method for testing behavior that would otherwise be too dangerous for the participant, such as crossing streets (Bowman & Liu, 2017; Thévin, Briant, & Brock, 2020; Rachitskaya et al., 2020) or driving with low vision (Alberti et al., 2014). On the other hand, AR headsets can be used in real-life situations, rather than virtual environments. AR can also be used as an accessibility tool, such as enhancing text (e.g., Huang et al., 2019; Zhao, Geng, et al., 2017; similar to VR) or, more notably, highlighting obstacles while navigating a real environment (e.g., Hicks et al., 2013; van Rheede et al., 2015).

Both AR and VR technologies afford the ability to simulate prosthetic vision without the need for invasive surgical procedures (Xia, Hu, & Peng, 2015; Sanchez Garcia et al., 2020; Thorn et al., 2020). By first simulating different implants and augmentation strategies in VR, theoretical predictions can potentially be tested in high-throughput experiments with sighted participants acting as “virtual patients” (Kasowski et al., 2021; Beyeler & Sanchez-Garcia, 2022). This may drastically speed up the development process of new prosthetic implants.

Desktop monitors were another trusted device type with constant interest over the years (see Supplementary Materials). Monitors can be particularly useful if they are used as a gaze-contingent display to study changes in eye movements (Table 2). Nonelectronic wearables (glasses, goggles, etc.) were the inexpensive but third-most common option in our dataset (e.g., Wood, Chaparro, Carberry, & Chu, 2010; Copolillo et al., 2017; Alberti & Bex, 2018).

With many exciting BLV applications in development, a person with low vision might wonder which technology to choose for their own good. Whereas our review has highlighted the relative benefit of visual feedback over audio in several places (Strumillo, 2010; Zhao et al., 2020), participants in these studies frequently requested multimodal feedback, which is consistent with other recent reviews on the subject (Santos et al., 2021; Creem-Regehr, Barhorst-Cates, Tarampi, Rand, & Legge, 2021). Another consideration is the cost of these accessibility aids, which is the most cited barrier to existing accessibility technologies even in high-income countries (UNICEF, 2022). As “low vision” encompasses such a heterogeneous demographic of people with different accessibility needs and individual preferences, the answer may have to be highly subjective as well.

### The experimental tasks studied with XR

For both low and prosthetic vision, visual search and recognition tasks were the most common (*n* = 114), followed by spatial cognition tasks related to orientation and mobility (*n* = 78) and low-level visual function testing (*n* = 35). While acuity is known as the standard for assessing visual function, the degree to which it associates to other tasks still remains to be explored, especially for changes in acuity with simulated conditions. Future work would benefit from comparing performance across tasks not limited to low-level visual function. Future development of AR smartglasses could potentially improve residual visual function for a range of tasks rather than one.

With the exception of low-vision augmentation studies, it is worth noting that most work involved sighted participants viewing SLV (*n* = 95 out of 166) or SPV (*n* = 55 out of 61). While this may reflect difficulty recruiting BLV participants, simulations have so far proved a valuable tool to enable large-scale behavioral studies and the quick prototyping of novel augmentation strategies.

### Key challenges and scientific gaps

XR technologies have seen major improvements in functionality and costs over the past decade, and the interest to use these devices for blindness and low-vision research has risen accordingly.

However, there are a number of challenges and limitations that were common across the different research areas:

• It is unclear to what extent simulations of low vision and prosthetic vision match the visual experience of people with BLV. Only a few studies thoroughly grounded their simulations in real patient data (e.g., Jones et al., 2020; Thorn et al., 2020), and studies using simulations had much younger participants than the target BLV group (μ = 29.2 and μ = 59.72 years, respectively). Many studies used crude approximations of the underlying eye condition and ignored the immersiveness of their simulation. For example, only 40% of studies used a gaze-contingent display, meaning that sighted participants could artificially increase their field of view through eye movements. Ignoring this aspect could result in simulated participants performing much better on tasks than those with the condition being studied. Addressing this could lead to more insightful simulations.
• While many studies (*n* = 76) used BLV participants to test the performance of a new system, very few studies consulted with the BLV community (*n* = 46) during the early phases of their study. Instead, the majority of studies focused on technical developments such as exploring different computer vision and enhancement techniques (e.g., Hommaru & Tanaka, 2020; van Rheede et al., 2015; McCarthy et al., 2015) and reporting quantitative measures such as mobility efficiency and errors, obstacle detection rates, and clinical visual measurements (Houston et al., 2018; Hicks et al., 2013; Barhorst-Cates, Rand, & Creem-Regehr, 2017). Less emphasis has been placed on understanding the usability and suitability of these aids in people with different levels of residual vision and underlying conditions, or whether or not these accessibility aids address the information needs of BLV users.
• Even fewer studies (*n* = 31) collected BLV participants’ opinions after the study. While the proposed systems may have improved performance in specific tasks, the systems must also be user-friendly and avoid steep learning curves. Struggling to adapt to new technologies may limit device use or prevent end users from acquiring the necessary skill set to fully utilize a new accessibility aid. Surveying user preferences at the end of a performance evaluation could lead to insights that may increase usability and adoption rates.

Unfortunately, to the best of our knowledge, none of the reviewed devices and applications have found widespread adoption. While most reviewed XR technologies are still in the development phase, it is interesting to note that currently available low-vision aids have a poor adoption rate as well. A recent survey highlighted several potential issues that include social stigma, low usability, and high cost (Sivakumar et al., 2020). Some also cited low awareness of available technologies. It is our opinion that at least some of these issues can be addressed by involving low-vision users in the decision-making and development during every step of the design process (Beyeler & Sanchez-Garcia, 2022), in a practice known as human-centered design (Rubin & Chisnell, 2011). Many studies in our collection seem poised for success in the near future (e.g., Hwang & Peli, 2014; McCarthy et al., 2015; Zhao, Cutrell, et al., 2019), and we are hopeful that addressing the highlighted gaps in the existing literature will lead to increased usability and adoption of different XR-based accessibility aids.

## Conclusion

In conclusion, our systematic review has highlighted the benefits of XR technology for BLV research, but challenges still remain. By broadening end-user participation to early stages of the design process and shifting the focus from behavioral performance to qualitative assessments of usability, future research has the potential to develop XR technologies that may not only allow for studying vision loss but also enable novel visual accessibility aids with the potential to impact the lives of millions of people living with vision loss.

## Supplementary Material

Supplement 1

Supplement 2
