# Supplementary material for: A systematic review of extended reality (XR) for understanding and augmenting vision loss
Source: J Vis. 2023 May 4;23(5):5. doi: 10.1167/jov.23.5.5 (PMC10166121; doi:10.1167/jov.23.5.5)
Supplement: Supplement 1 [file jovi-23-5-5_s001.pdf]

# A Systematic Review of Extended Reality (XR) for Understanding and Augmenting Vision Loss

Justin Kasowski[1,*], Byron A. Johnson[2,*], Ryan Neydavood[2], Anvitha Akkaraju[2], and Michael Beyeler[2,3]

[1]Graduate Program in Dynamical Neuroscience, University of California, Santa Barbara, CA, 93106 [2]Department of Psychological & Brain Sciences, University of California, Santa Barbara, CA, 93106 [3]Department of Computer Science, University of California, Santa Barbara, CA, 93106 [*]These authors contributed equally

Words: 9045

Commercial interests: None.

Funding: This work was partially supported by NIH DP2-LM014268 to M.B.

CONTACT Justin Kasowski. Email: justin_kasowski@ucsb.edu

## Appendix A. Publications by Venue

Upon completion of the systematic review, we identified 227 papers from 105 different venues. 57 of these were conference publications (Table A1) that included full papers, workshop papers, and extended abstracts/short papers. The majority of papers were classified as full papers by their respective venue. Of the 35 different conference venues in our dataset, the most popular conference was the Annual Meeting of the IEEE Engineering in Medicine and Biology Society (EMBC) followed by ACM ASSETS and CHI. Not surprisingly, the range of conference venues also included top-tier conferences in mixed reality (e.g., IEEE VR, ISMAR, and UIST), accessible technology (ICCHP, UAHCI), and ubiquitous computing (e.g., UbiComp, PerCom, ACIIW). There were also a number of publications that were considered short papers or extended abstracts that accompanied a poster presentation or demo. A small fraction of papers were part of a workshop or satellite event instead of the main conference track.

| Venue | Full | Short | Workshop | Total |
|---|---|---|---|---|
| IEEE EMBC | 11 | 0 | 0 | 11 |
| ACM ASSETS | 4 | 2 | 0 | 6 |
| ACM CHI | 4 | 1 | 0 | 5 |
| ICCHP | 2 | 0 | 0 | 2 |
| IEEE VR | 0 | 0 | 2 | 2 |
| CVPR | 0 | 0 | 2 | 2 |
| All others | 21 | 6 | 2 | 29 |
| **Total** | **42** | **9** | **6** | **57** |

Table A1. Conference publications by venue

The other 170 publications were full-length articles that appeared in one of 70 different scientific journals (Table A2). Here, the largest body of work appeared in vision science journals that specialize in either basic (e.g., Journal of Vision, Vision Research) or clinical research (e.g., Optometry & Vision Science, Investigative Ophthalmology & Visual Science, Translational Vision Science & Technology). A number of papers also appeared in biomedical engineering journals (e.g., Journal of Neural Engineering, IEEE Transactions) and general-purpose journals (e.g., PLOS ONE, Scientific Reports).

| Venue | Count |
|---|---|
| Optometry & Vision Science | 13 |
| Vision Research | 13 |
| Journal of Neural Engineering | 12 |
| Journal of Vision | 12 |
| Ophthalmic & Physiological Optics (OPO) | 11 |
| Artificial Organs | 8 |
| PLOS ONE | 8 |
| Translational Vision Science & Technology (TVST) | 8 |
| Investigative Ophthalmology & Visual Science (IOVS) | 6 |
| All others | 79 |
| **Total** | **170** |

Table A2. Journal publications by venue

## Appendix B. Publications by Year

Additionally, we wanted to know how the field has progressed over the last decade. Fig. B1 summarizes the number of studies for each of our four major groups. We found that the overall number of papers in the corpus has been steadily increasing over the last decade, largely driven by increasing interest in extended reality (XR) for low vision (augmentation: 500.0% increase, perception: 141.7% increase since 2010).

| Year | 2010-11 | 2012-13 | 2014-5 | 2016-17 | 2018-19 | 2020-21 | **Total** |
|---|---|---|---|---|---|---|---|
| Low vision: | | | | | | | |
| - XR for studying perception & behavior | 12 | 10 | 11 | 12 | 16 | 29 | **90** |
| - XR for augmenting low vision | 4 | 4 | 8 | 15 | 21 | 24 | **76** |
| Blindness: | | | | | | | |
| - XR for studying perception & behavior | 5 | 2 | 8 | 5 | 4 | 3 | **27** |
| - XR for augmenting prosthetic vision | 4 | 6 | 6 | 5 | 7 | 6 | **34** |
| **Total** | **25** | **22** | **33** | **37** | **48** | **62** | **227** |

Table B1.Number of publications per year and application area.

Interestingly, the recent rise in popularity of XR technologies for blindness or low vision (BLV) can be primarily attributed to virtual reality (VR) and augmented reality (AR) wearables (Table B2), despite an increase in availability of handheld devices such as smartphones and tablets. Research in VR wearables increased by 112.5% from 2010–11 to 2020–21, and monitor-based XR research increased by 433.3%.

| | 2010-11 | 2012-13 | 2014-15 | 2016-17 | 2018-19 | 2020-21 | Total |
|---|---|---|---|---|---|---|---|
| Monitors | 3 | 7 | 8 | 9 | 9 | 16 | **52** |
| Handheld devices | 0 | 1 | 1 | 4 | 4 | 4 | **14** |
| Nonelectronic wearables | 12 | 6 | 7 | 6 | 11 | 5 | **47** |
| VR wearables | 8 | 5 | 9 | 7 | 9 | 17 | **55** |
| AR wearables | 2 | 3 | 8 | 11 | 15 | 20 | **59** |
| **Total** | **25** | **22** | **33** | **37** | **48** | **62** | **227** |

Table B2. Number of publications per year and device type.
